# Half a century of bacteriophage lambda recombinase: In vitro studies of lambda exonuclease and Red‐beta annealase

**DOI:** 10.1002/iub.2343

**Published:** 2020-07-03

**Authors:** Jodi L. Brewster, Gökhan Tolun

**Affiliations:** ^1^ Molecular Horizons and School of Chemistry and Molecular Bioscience University of Wollongong Keiraville New South Wales Australia; ^2^ Illawarra Health and Medical Research Institute Wollongong New South Wales Australia

**Keywords:** annealase, exonuclease, phage lambda, Red‐beta, single‐stranded DNA‐binding protein, two‐component recombination

## Abstract

DNA recombination, replication, and repair are intrinsically interconnected processes. From viruses to humans, they are ubiquitous and essential to all life on Earth. Single‐strand annealing homologous DNA recombination is a major mechanism for the repair of double‐stranded DNA breaks. An exonuclease and an annealase work in tandem, forming a complex known as a two‐component recombinase. Redβ annealase and λ‐exonuclease from phage lambda form the archetypal two‐component recombinase complex. In this short review article, we highlight some of the *in vitro* studies that have led to our current understanding of the lambda recombinase system. We synthesize insights from more than half a century of research, summarizing the state of our current understanding. From this foundation, we identify the gaps in our knowledge and cast an eye forward to consider what the next 50 years of research may uncover.

**Abbreviations**
AFM: atomic force microscopy
CTD: C‐terminal domain
dsDNA: double‐stranded DNA
EM: electron microscopy
nt/s: nucleotides/second
NTD: N‐terminal domain
Phage λ: bacteriophage lambda
SSA: single‐strand annealing homologous DNA recombination
SSB: single‐stranded DNA‐binding protein
ssDNA: single‐stranded DNA
λExo: λ‐exonuclease

## 1 INTRODUCTION

Single‐strand annealing homologous DNA recombination (SSA) is a way in which double‐stranded DNA (dsDNA) breaks can be repaired. Breaks in dsDNA can occur in a variety of ways: due to endonucleases, at chromosomal crossovers, at stalled or reversed replication forks, or due to environmental agents such as chemicals and radiation.^1^ SSA has been adopted ubiquitously by dsDNA viruses and exploited to have key roles in DNA replication, evasion of host defenses, and generation of genetic diversity. Bacteriophage lambda (phage λ) is a dsDNA virus that infects the bacterium *Escherichia coli*. Phage λ encodes its own two‐component recombinase for catalyzing SSA, which is very efficient^2^ and has been widely studied as the model system for SSA. The efficiency of the λ Red recombination system has also led to its adoption as a tool for genetic manipulation, principally recombineering, which can be used to introduce deletions, insertions, and point mutations in DNA (reviewed in Reference 3).

The **re**combination‐**d**eficient, Red, system of phage λ is named for the phenotype exhibited by mutants of its constituent genes: *exo* (Redα/λ‐exonuclease [λExo]), *bet* (Beta/ Redβ), and *gam* (Gam/γ‐protein). The λExo protein binds to dsDNA ends, resecting one strand in the 5′ to 3′ direction to expose a 3′ single‐stranded DNA (ssDNA) overhang. Redβ protein binds to this nascent ssDNA, promoting annealing to a complementary or homologous strand while protecting it from nucleolytic degradation. The two proteins associate, forming a two‐component recombinase complex working in tandem to mediate recombination (reviewed in Reference 4). Although replication and recombination are adversely affected in *gam* mutants *in vivo*,^5^ there is no evidence that Gam interacts with the phage λ recombination machinery, and therefore Gam will not be a focal point of this review.

**FIGURE 1:**
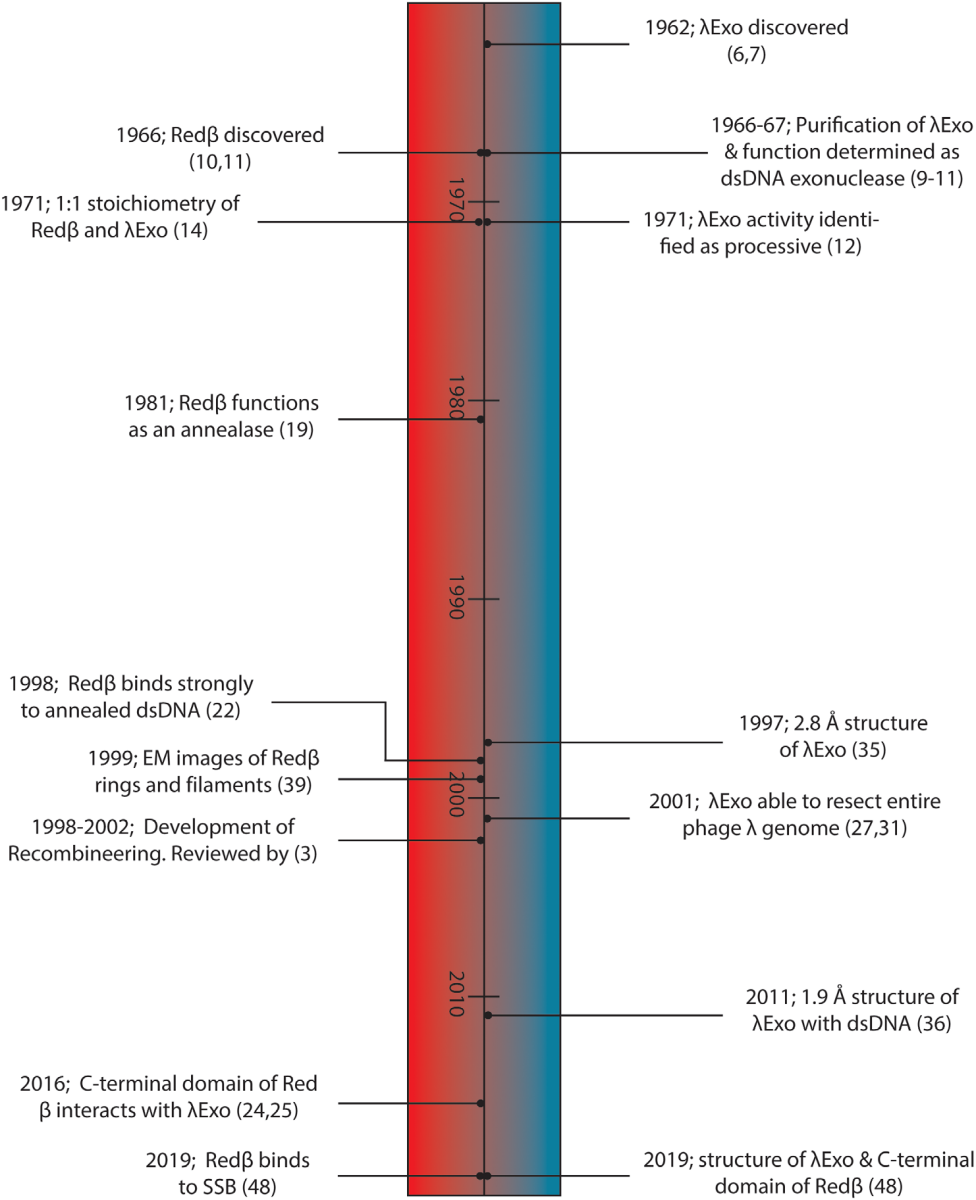
Timeline of λExo and Redβ discovery and characterization

The λ recombinase system has been studied for over 50 years and a daunting body of research has been amassed over this period. Herein, we attempt to summarize some of the key *in vitro* experiments (Figure 1) that have led to our present understanding of λ recombination. At present, however, this knowledge is far from comprehensive and there are many questions yet to be answered.

## 2 A BRIEF HISTORY OF LAMBDA RECOMBINASE

Primed by the publication of Watson and Crick's double helix structure of DNA in 1953, the decades of the 1960s, 1970s, and 1980s ushered in a golden age in the advancement of molecular biology research. During this period, *E. coli* and phage λ were both widely studied as model organisms, leading to the development of many genetic manipulation and molecular cloning techniques. From this foundation, phage λ also emerged as a model system for virology and investigation of the balance and genetic switch between the lytic and lysogenic cycles. λExo was discovered in 1962^6^, ^7^ when an increase in exonuclease activity was observed upon chemically inducing *E. coli* lysogens into the λ lytic cycle. λExo synthesis was one of the earliest detectable events, associated with the disappearance of the lytic repression system. This exonuclease had an alkaline pH optimum of 9.5^6^, ^8^ and a requirement for divalent magnesium^8^, ^9^ *in vitro*. A few years later in 1966, an enriched preparation of λExo was obtained from an overexpressing mutant strain (λ T_11_), and this also led to the discovery of Redβ, which co‐purified with λExo.^9^, ^10^

### 2.1 An unusual exonuclease

**FIGURE 2:**
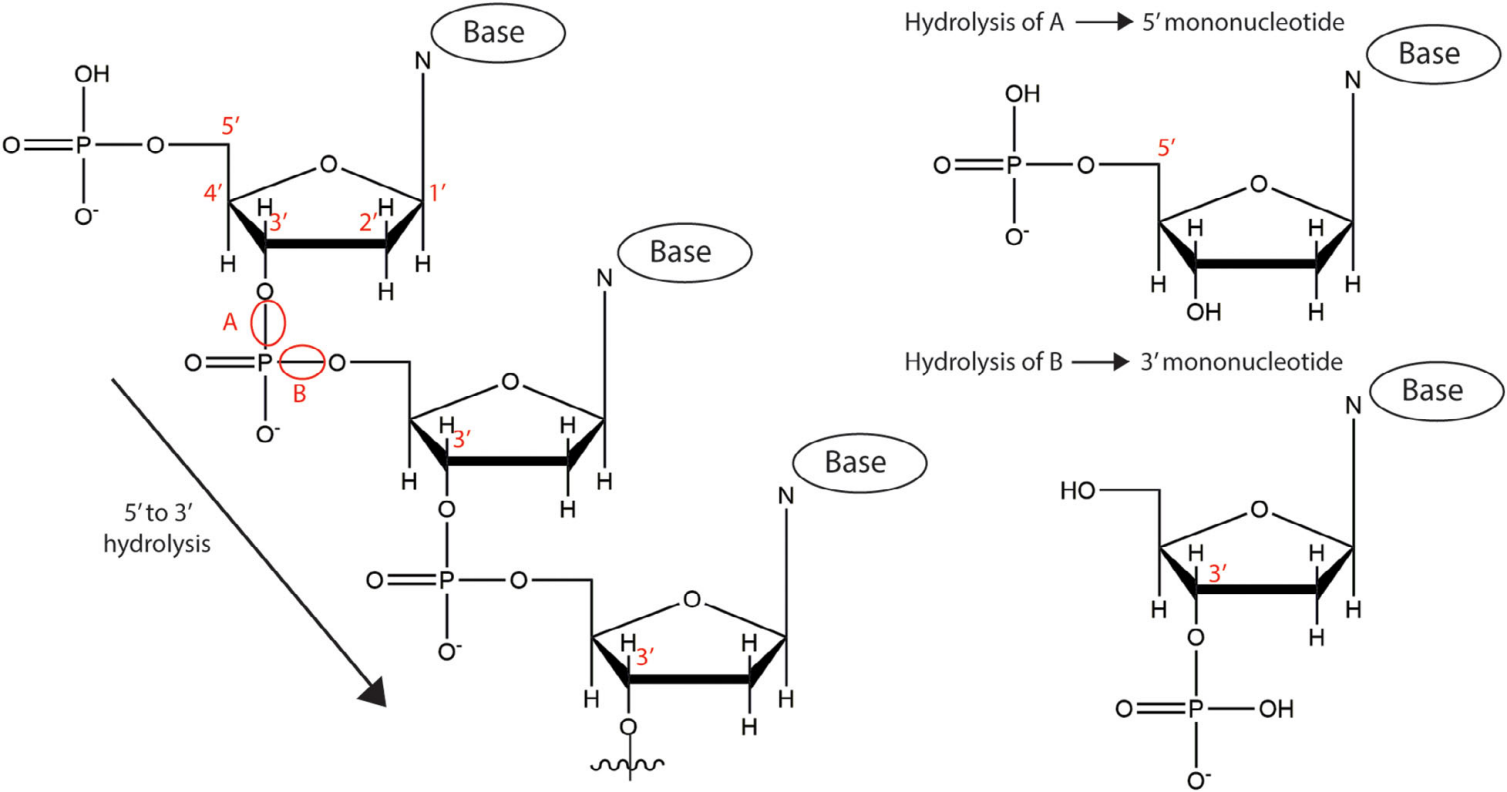
DNA hydrolysis by λExo to release 5′ mononucleotides. A 3′ mononucleotide is shown for comparison

The following year, in 1967, λExo was purified by crystallisation^8^ allowing for its functional and kinetic characterization. The enzyme was found to have an uncommon exonuclease activity: 5′ to 3′ directionality that liberated 5′ mononucleotides (i.e., with a phosphoryl group on the sugar 5′‐carbon) (Figure 2).^11^ Most exonucleases characterized at the time operated in the opposite (3′ to 5′) direction and this was the first report of a rare subset. In accordance with this, λExo also displayed a marked specificity for phosphorylated rather than hydroxylated 5′ DNA termini.^11^ The structural basis for this specificity would come to light decades later with the first structure of λExo and would prove to be integral to understanding its catalytic mechanism (see in the following).

As another point of difference, λExo showed a strong specificity for dsDNA, concurrently attacking both strands from their 5′ termini. Other 5′ to 3′ exonucleases degraded dsDNA and ssDNA at approximately equivalent rates. However, λExo was found to degrade dsDNA up to 350 times faster than ssDNA and showed barely detectable rates of hydrolysis of RNA.^11^ Further investigation into its substrate specificity indicated that it was unable to initiate hydrolysis at a nick within dsDNA. This was true for nick sites in both linear dsDNA and Hershey circles^12^—the circularized phage λ genome formed by self‐annealing of the two 5′ ssDNA overhangs.^12^ Although λExo could bind weakly to such structures, digestion was initiated at neither nicks nor gaps.^12^, ^13^ In addition, binding of λExo to dsDNA was unaffected by the presence or absence of Redβ and the rate of hydrolysis at 25°C was calculated as ~4 nucleotides/second (nt/s).^13^ At this time, the monomeric molecular weights of λExo and Redβ were first estimated to be 24 and 28 kDa (close to the actual values of 25.9 and 29.7 kDa, respectively), and a subunit stoichiometry of 1:1 was predicted for their complex.^14^

### 2.2 The first model for Red recombination

In 1970, Red DNA recombination was studied through complementation experiments to understand the components involved.^15^, ^16^ Various *red* point mutant and deletion strains were crossed, and the mutants were assigned into groups based on the occurrence of recombination. The positions of *exo* and *bet* were genetically mapped adjacent to one another on the phage λ genome and it was shown that both were required for recombination.^15^, ^16^ With the roles of λExo and Redβ thus pinpointed to recombination, studies turned to investigating the underlying mechanism.

**FIGURE 3:**
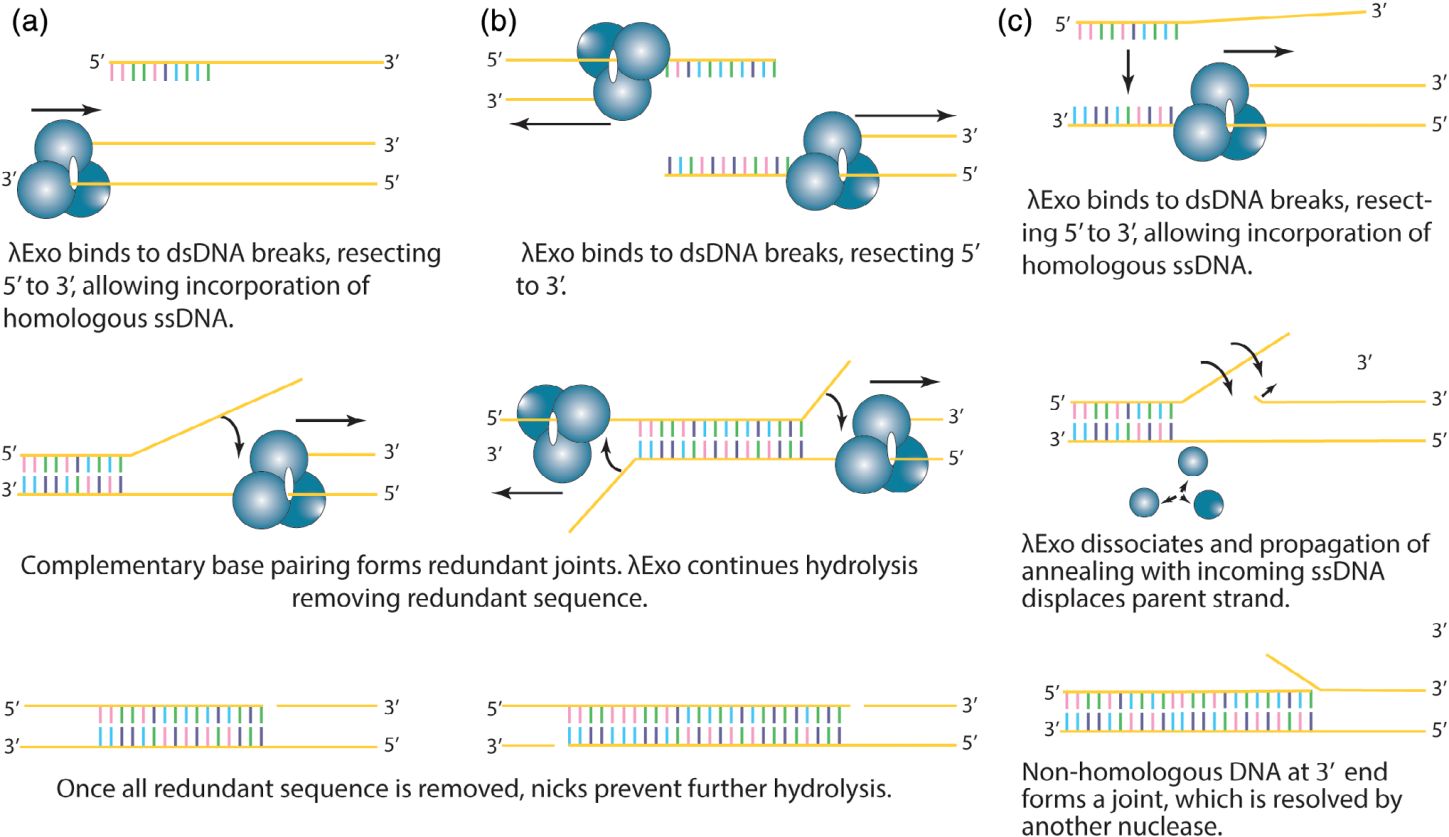
Early models for Red recombination. (a) Single‐strand assimilation.^13^ (b) Single‐strand assimilation to remove redundant joints.^17^ (c) Strand exchange resulting in joint formation,^18^ resolved by a 5′ to 3′ exonuclease or the flap endonuclease activity of DNA polymerase I. λExo is shown as cyan spheres. Figures are adapted from the references cited

The experiments of Carter and Radding^12^, ^13^ showed that once digestion of dsDNA begins with stoichiometric amounts of substrate and enzyme, the subsequent addition of more dsDNA substrate did not slow the enzymatic rate. This observation indicates that rapidly reversible interaction between the enzyme and its substrate does not occur, as required for Michaelis–Menten kinetics. Instead, it suggests that λExo remains bound to the dsDNA, and that hydrolysis continues until complete digestion of the DNA segment (3 kb was measured).^12^ Therefore, λExo acts as a processive enzyme,^12^ a realization that when combined with the observation that it is not active at nicks led to the single‐strand assimilation model.^13^ The authors proposed that rather than an inability to initiate hydrolysis, weak binding of λExo at nicks instead represented the completion of branch digestion and was a mechanism for the removal of redundant joints (Figure 3a).

The model was swiftly refined as a means to affect homologous recombination,^17^ whereby λExo binds to dsDNA termini, catalyzing 5′ end resection to expose regions of ssDNA to which Redβ binds. Complementary strands of ssDNA then hybridize while resection continues to remove the redundant sequence, assimilating the two new strands (Supplementary Animation 1). λExo stalls when it reaches a nick, gap, or the end of the DNA strand, stopping digestion and dissociating from the DNA (Figure 3b). This model for redundant joint removal could also incorporate strand exchange, with λExo dissociating and the annealing incoming strand displacing the parent strand to give rise to a branch point (Figure 3c). The role of Redβ in this mechanism was not clear, but it was presumed to be physically associated with λExo. However, as joint repair appeared to be as efficient with λExo alone, Redβ was thought to play no part in the reaction.^18^

### 2.3 The overlooked Redβ enters the spotlight

The breakthrough in elucidating the role of Redβ did not come until the early 1980s, 15 years after it was discovered to co‐purify with λExo.^9^, ^10^ It was found to be able to renature complementary DNA strands *in vitro*.^19^ Furthermore, lysogens with mutations in the *bet* gene were recombination defective, showing that Redβ is essential for phage λ recombination *in vivo*. The function of Redβ was subsequently refined when it was able to be purified away from λExo.^20^ Redβ was found to exhibit some properties common to both the RecA recombinase and single‐stranded DNA‐binding proteins (SSBs) from *E. coli*.^20^ Redβ alone could not promote heteroduplex joint formation (dsDNA recombination), but was able to catalyze the annealing of complementary single strands, such as the *cos* (cohesive) ends of the linear λ chromosome,^20^ which are both functional characteristics shared with RecA, albeit RecA utilizes ATP and Redβ does not.^19^ However, unlike RecA but in a manner reminiscent of SSB, Redβ destabilized secondary structure in ssDNA, allowing it to bind and protect ssDNA (but not dsDNA) from digestion by DNase.^20^

A decade later, the DNA‐binding properties of Redβ were examined in detail. It was determined that it bound to ssDNA with a *K*_D_ of 1.8 μM^21^ and although it did not bind to duplex DNA (consistent with previous observations^20^), it remained strongly bound to the renatured dsDNA product of an annealing reaction with complementary ssDNA oligonucleotides.^22^ These binding characteristics were hypothesized to model a mechanism of action for Redβ whereby the protein binds to ssDNA and interaction with a complementary strand results in an annealed nucleoprotein complex. The higher affinity of Redβ for DNA in this state in turn stabilizes the dsDNA product.^22^ What drives the eventual dissociation of Redβ from the reannealed product remains an open question. Further characterization of the interaction of Redβ with ssDNA established a binding footprint of five nucleotides per monomer,^21^ which was consistent with a previous prediction of four nucleotides.^20^ It was also shown that Redβ required a minimum of 28–36 nucleotides to form a stable nucleoprotein complex, though this has subsequently been refined to just 23 nucleotides *in vivo*.^23^ Finally, through crosslinking Redβ to a 36mer oligonucleotide, DNA‐binding was localized to a 20 kDa N‐terminal domain (NTD).^21^

In 2016, 10 years after identification of the NTD of Redβ as the DNA‐binding domain, Smith and Bell^24^ and Subramaniam *et al*.^25^ independently investigated the functional roles of the N and C‐terminal domains (CTDs) in detail. Smith and Bell^24^ examined the effect of DNA length on the binding affinity for WT and C‐terminally truncated (residues 1–177) Redβ. They found that binding affinity was largely independent of DNA length beyond the 36‐nucleotide threshold.^21^ The DNA‐binding characteristics of full‐length Redβ were also consistent with previous reports^21^, ^22^ with *K*_D_ ~ 1.6 μM for ssDNA and a threefold higher affinity for annealed dsDNA (*K*_D_ ~ 0.5 μM).^24^ However, the opposite was true for C‐terminally truncated Redβ (*K*_D_ ~ 0.6 and 1.2 μM, respectively). Attention was then turned to the CTD of Redβ, with circular dichroism spectroscopy experiments by Smith and Bell^24^ predicting that residues 182–261 formed a predominantly α‐helical fold, which was sufficient to form a stable interaction with λExo. Subramaniam *et al*.^25^ reported a *K*_D_ of ~8 μM for the interaction between full‐length Redβ and λExo, but also found it was weakened by deletion to the NTD domain, suggesting that the NTD may still have some influence on λExo binding, and hence this point requires further clarification. Both groups then went on to show that while still able to affect annealing, C‐terminally truncated Redβ was unable to mediate recombination^24^, ^25^ and that the removal of just 11 amino acids from the C‐terminus^24^ or the introduction of point mutations^25^ prevented recombination. However, Smith and Bell^24^ hypothesized that abolition of recombination was not due to disruption of the Redβ (via the CTD)–λExo interaction. λExo is thought to be required to load Redβ onto the 3′ overhang for dsDNA recombination, a function that may be disrupted by the loss of the CTD. However, Redβ‐mediated ssDNA recombination, which is independent of λExo, was also affected.

## 3 THE NEXT GENERATION OF KINETIC AND MECHANISTIC STUDIES—SINGLE‐MOLECULE CHARACTERIZATION

The first time λExo was studied at the single‐molecule level was in 1999 and an average rate of digestion of 15–20 nt/s was calculated.^26^ This was consistent with values obtained by previous ensemble studies (see above)^12^, ^13^ and would also be the consensus of later single‐molecule studies (12–32 nt/s).^27^, ^28^, ^29^ However, two other studies by Matsuura *et al*.^30^ and Oliver‐Calixte *et al*.^31^ reported much higher catalytic rates of approximately 1,000 nt/s. Oliver‐Calixte *et al*. suggested that the high rate they observed may have been an artifact of an experimental design that was common to both studies—directly tethering the enzyme to a solid support may have resulted in increased λExo stability.^30^, ^31^ However, this wide discrepancy in reported rates remains unresolved.

The benefits of using single‐molecule techniques to study this system were realized with the discovery of pauses inherent in λExo progression. In these experiments, dsDNA substrates were tethered between a coverslip and a bead in an optical trap, and the conversion of dsDNA to ssDNA was measured in real time by taking advantage of the difference in their contour lengths at low stretching forces.^29^ Pausing behavior had previously been observed with processive DNA replication enzymes, but the mechanism and significance were not well understood. Though apparently strand‐specific and sequence‐dependent in nature, the pauses were also stochastic. Pause probability and duration varied widely with most being shorter than 5 s, but some longer than 30 s were also recorded.^29^ It was also observed that λExo could escape the pause by diffusion‐based backtracking.^32^ The strongest pauses occurred immediately prior to a GGCGATTCT sequence with GGCGA identified as the crucial motif.^29^ The GGCGA pause motif is found in the left *cos* (*cosNL*) site of the λ chromosome and Perkins *et al*. speculated that it may slow digestion from this end of the DNA *in vivo*. They further concluded that this was consistent with early observations of lowered replication‐independent (i.e., Red‐dependent) recombination frequencies.^33^ Nevertheless, Perkins *et al*. were able to further reduce the pause location to the first guanosine of the motif, which led them to propose that λExo translocation and DNA strand separation occur in a stepwise manner, a single base pair at a time.^29^

van Oijen *et al*.^27^ used a similar strategy to study λExo kinetics by stretching bead‐tethered dsDNA substrates in a hydrodynamic flow and imaging the bead position. This very effectively allowed for the multiplexing of measurements, greatly increasing experimental throughput whilst retaining the major advantages of single‐molecule measurements. By this method, an average processivity of 18 ± 8 kb of resection was observed. Furthermore, in approximately 8% of cases, the full 48.5 kb of the phage λ substrate was digested without dissociation,^27^, ^31^ a truly remarkable observation. This level of processivity is much higher than that seen in early experiments^12^ and may have been due to the use of much longer DNA substrates. However, the crucial outcome of this study was observation of a sequence‐dependent effect on rate. It suggested that base‐pair melting was the rate‐limiting step in the catalytic cycle^27^ which was consistent with the high GC content of the long pause sites discovered by Perkins *et al*.^29^ Though it was not experimentally determined, it was reasoned that base‐pair melting must occur prior to hydrolysis of the scissile phosphoester bond. For the first time, this gave rise, if not to a mechanism, at least to a probable sequence of catalytic events. Thus, base‐pair melting involving the disruption of hydrogen bonds and base stacking interactions is followed by hydrolysis of the phosphoester bond, which drives translocation to the next nucleotide.^27^ Repeated catalytic cycles that underpin λExo processivity were proposed to be a function of competing forward and backward translocation modes, with the probability of each mode dictated by catalytic efficiency (*k*_cat_), directional rate constants, and the relative affinity of the enzyme for the dsDNA substrate versus the ssDNA product.^28^

## 4 STRUCTURAL REVELATIONS ON RED RECOMBINATION

It was some time before structural information on λExo and Redβ was available to shed more light on the relationship between the two proteins and the mechanism by which they affected homologous recombination. λExo was crystallized in 1985, though the crystals diffracted only weakly to 6 Å resolution and the structure could not be solved.^34^ It took more than another decade, but finally in 1997, the structure was solved to 2.4 Å.^35^ The homo‐trimeric λExo is toroidal in shape.^35^ The funnel‐like central channel tapers from ~30 Å at one end to ~15 Å at the other, permitting the entry of linear dsDNA but ensuring that only ssDNA can be extruded. The quaternary structure provided insight into many of the experimentally determined properties of λExo. The symmetrical arrangement of the monomers in the toroid equivalently orientates all three active sites, ensuring unidirectionality of hydrolysis. Loading of the trimer also explained why a dsDNA end with a short 3′ ssDNA overhang is the preferred substrate. Furthermore, once loaded, the enzyme would remain on the DNA substrate until either dissociation of the trimer or the strand‐end, consistent with the processivity of λExo catalysis.

**FIGURE 4:**
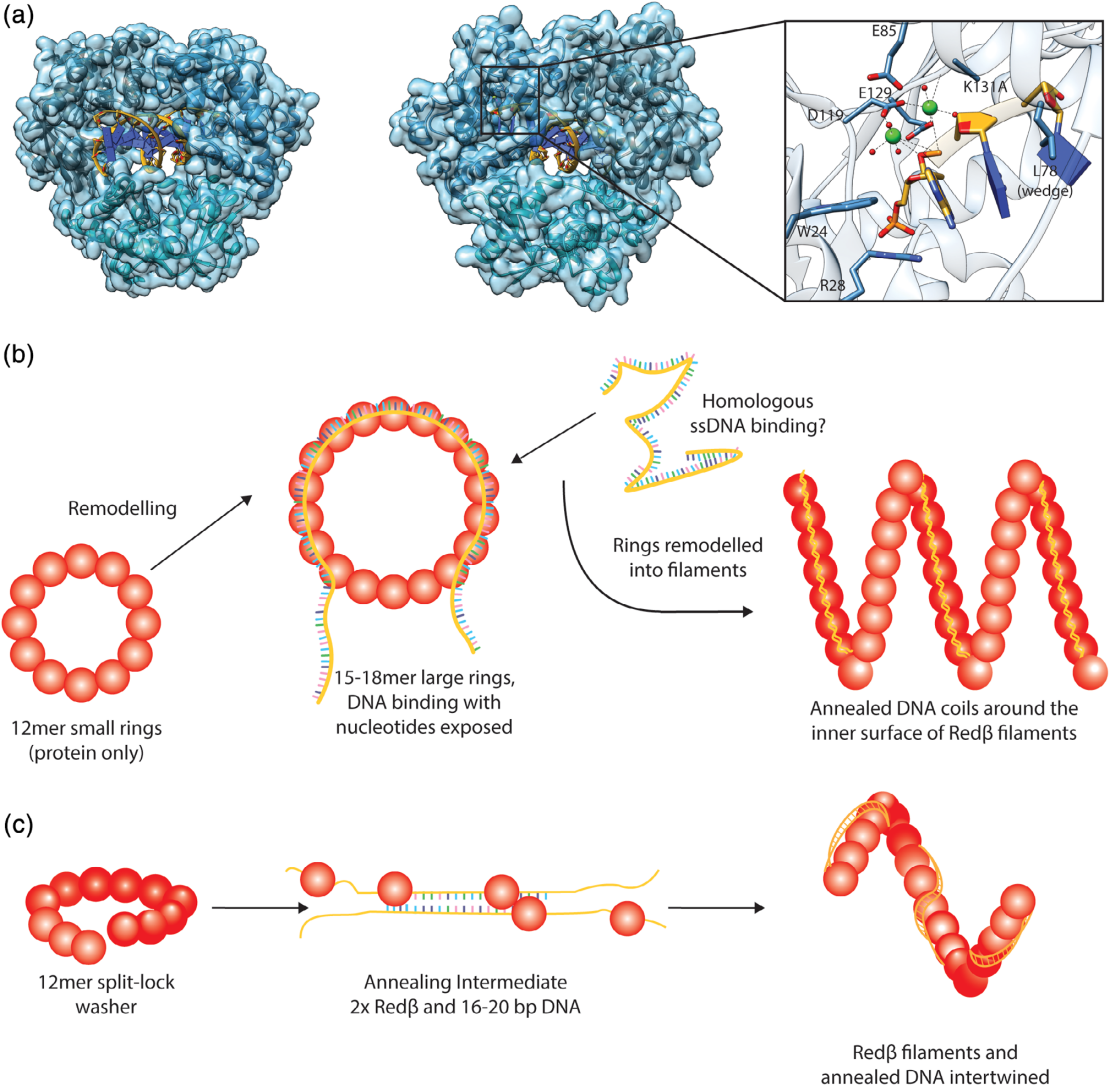
Structures of λExo and Redβ. (a) λExo trimer with DNA bound (PDB ID: 3SM4), from Reference 36. Left—dsDNA entering the active site; Center—ssDNA leaving the active site; Right—Active site showing magnesium ions (green spheres) coordinating water molecules (red spheres) and key residues. (b, c) Schematic representations of the Redβ super‐structures. Rings remodeled into filaments, based on the model presented by Passy et al.^39^ and the crystal structure of Rad52 with ssDNA (PDB ID: 5XRZ)^45^ (b); and split‐lock washers remodeling into filaments via an annealing intermediate^46^, ^47^ (c)

In 2011, a higher resolution (1.9 Å) crystal structure of λExo in complex with DNA (Figure 4a) was reported.^36^ Mutation of the catalytic base (Lys131Ala) trapped a stable enzyme‐DNA complex, revealing additional details underlying the mechanism. The structural details and proposed mechanism have been discussed in depth in a recent review by Caldwell and Bell.^4^ Briefly, the crystal structures support an “electrostatic ratchet” mechanism for the processive activity of λExo.^36^ The DNA duplex is positioned by binding the 5′ phosphate into a positively charged pocket, with Trp24 and Arg28 making hydrogen bond contacts, which explained the specificity of the enzyme for 5′ phosphorylated DNA substrates (see above).^11^ Correct positioning of the DNA in conjunction with a hydrophobic wedge, a non‐polar loop abutting the terminal nucleotide, drives the unwinding of DNA and separation of the Watson‐Crick base pairs (Figure 4a). This model agrees with previous single‐molecule behavior measured by van Oijen *et al*.,^27^ who suggested that base‐pair melting is the rate‐limiting step of the reaction. Finally, an interaction was discovered downstream of the active site, with Arg45 inserting into the minor groove of the double helix, which is proposed to maintain alignment of the DNA‐enzyme complex.^36^

The electrostatic ratchet model was systematically validated through mutagenesis of key residues in the active site (Glu85, Asp119, and Lys131), the central channel of the toroid (Arg45, Lys49, Met53, Lys76, and Arg137), the phosphate‐binding pocket (Trp24, Arg28), and the hydrophobic wedge (Leu78).^37^ For each mutation, *in vitro* enzyme activity, dsDNA‐binding affinity, and *in vivo* recombination efficiency were comprehensively analyzed. It was found that *in vivo* recombination efficiency mirrored *in vitro* λExo activity. Three of the channel mutants retained moderate *in vitro* enzyme activity (~37–70% of wild‐type) and Arg45Ala retained nearly full activity, consistent with their proposed non‐catalytic roles. All other mutations either completely abolished or reduced *in vitro* enzyme activity to barely detectable levels. Conversely, the DNA‐binding affinity was most adversely affected in the channel mutants with Arg45Ala having the greatest effect. Changing Arg45 to lysine restored binding ability, highlighting the importance of the interaction between the positively charged side chain and the phosphate backbone of DNA.^36^, ^37^ Combined with the dependence of enzyme activity on an intact phosphate‐binding pocket, this places a greater mechanistic importance on electrostatic interactions than on the ratchet^37^—which likely has a greater effect on processivity than on catalytic turnover.

The role of Arg45 in maintaining the trajectory of λExo with respect to DNA raises the question of how the three active sites are utilized. Are the subunits used sequentially and if so, what determines the catalytic subunit? These questions were addressed by a “mutant poisoning” approach.^38^ Subunits of wild‐type and an inactive λExo mutant (Lys131Ala) were exchanged, resulting in the formation of hybrid trimers. The measured enzyme activity decreased approximately linearly with an increasing proportion of mutant protein present. From this, it was interpreted that the λExo subunits did not need to be used sequentially, as the inclusion of a single mutant subunit did not render the trimer inactive, manifesting as a sudden drop‐off in activity. However, trimers containing one or two mutant subunits were substantially less active than wild‐type trimers. This indicates that although activity is not dependent on sequential subunit processing, all three subunits are used in the course of strand digestion.^38^ It also does not exclude the possibility that sequential utilization occurs under conditions where all three subunits are active. Hence, further work is required to understand the fine details of λExo activity.

In 1999, just 2 years after the crystal structure of λExo was first solved, the electron microscopy (EM) ultrastructure of Redβ was published.^39^ Redβ was seen to exist in three different oligomeric states: small ~12 membered rings, larger ~15–18 membered rings and left‐handed helical filaments. In isolation, Redβ exclusively formed small rings. In the presence of ssDNA (short 30 nt oligonucleotides and longer M13 ssDNA), larger rings were predominant, but small rings and short helical filaments were also present. Large rings and filaments dominated in the presence of ssDNA generated by heat‐denaturing dsDNA, but filaments dominated with thermally reannealed dsDNA. The rings and filaments appeared to be dynamically related with evidence of super‐structural remodeling between forms, and filaments were often seen capped by rings.

Similar rings and filaments have also been seen with other annealases, such as the phage P22 Erf protein,^40^ RecT from *E. coli*,^41^ eukaryotic Rad52,^42^, ^43^ and ICP8 from HSV‐1.^44^ Passy *et al*.^39^ envisioned ssDNA to be wrapped around the outside of the large Redβ rings in a manner similar to that originally predicted for Erf.^40^ However, recent structural data for Rad52 suggest that ssDNA is bound in a groove in the outer surface of the protein rings,^45^ which would simultaneously remove secondary structure and position the nucleotides outward to facilitate annealing with complementary ssDNA.^39^, ^45^ Following annealing, it is proposed that the rings are remodeled into filaments. Unfortunately, the EM images did not provide direct evidence for the position of the DNA within the Redβ helices. But the density distribution observed suggested that dsDNA coils around the inner surface of the helix, protecting the annealed DNA from nucleolytic degradation^39^ (Figure 4b). Redβ protection of annealed DNA against digestion by DNase I, *E. coli* exonuclease I, and λExo had been experimentally shown the previous year.^22^

A decade later beginning in 2009, studies by the Stewart group using atomic force microscopy (AFM) led to the proposal of a revised model. Erler *et al*.^46^ showed that in the absence of DNA, what appeared to be rings at low resolution were resolved to be 11–12 subunit split lock‐washer circlets (a single turn of a shallow right‐handed helix; Figure 4c). It is possible that the small rings seen in the electron micrographs produced by Passy *et al*.^39^ represent the top view of a washer conformation. Alternatively, the washer may represent a different conformation. Though it is currently difficult to distinguish between these two possibilities, it should be kept in mind for future microscopy studies of annealases. It has also been proposed that the ring (or perhaps washer) conformations adopted by Redβ and other annealases are functionally important.^39^, ^40^, ^42^, ^43^ However, there is still debate over this due to conflicting results. Redβ was initially proposed to be dimeric in solution at a concentration of 2 mM,^21^ whereas the formation of ring‐like structures *in vitro* is proposed to occur at concentrations >8 μM.^39^, ^46^ However, both concentrations are supraphysiological, since the *in vivo* amount of Redβ is substantially lower at ~150 nM, at which it was reported to be mostly monomeric.^47^

Based on both AFM and single‐molecule data, the Stewart group have proposed a model in which monomeric Redβ binds randomly and non‐cooperatively to ssDNA, with annealing nucleated from sporadic intermolecular Redβ interactions. Redβ dimerization that initiates annealing leads to a conformational change that “clamps” the protein onto the DNA^47^ (Figure 4c). This complex was characterized as an annealing intermediate and identified as the minimal functional unit of recombination *in vivo*, comprising 16–20 bp of annealed DNA and two Redβ molecules.^46^ The models proposed by both Passy *et al*.^39^ and the Stewart group are then in agreement that annealing and polymerization of Redβ leads to the formation of stable left‐handed helical filaments. However, rather than the DNA duplex lining the inner surface of the helical filament as proposed by Passy *et al*.,^39^ the latter model proposes that the DNA and Redβ are intertwined, with dissociation possibly requiring helicase action.^46^, ^47^

In 2019, an X‐ray crystal structure of λExo in complex with the CTD of Redβ was published.^48^ The structure showed the point of interaction between the CTD of Redβ to be with α‐helix E of λExo. Based on structural homology of the CTD of Redβ to phage λ Orf protein, which binds to SSB, facilitating Rec‐mediated recombination, it was proposed that Redβ also binds to SSB.^48^ Subsequently, an interaction was validated between full‐length Redβ and a peptide consisting of the nine C‐terminal residues of SSB,^48^ which form a motif that interacts with a large number of other proteins.^49^ A modest *K*_D_ of ~10 μM was calculated, which was similar to that previously determined for Redβ binding to λExo (*K*_D_ of 7.9 μM).^25^ Though the similarity in the two binding affinities may be coincidental, binding interactions in the low micromolar range simultaneously allow for specific yet dynamic protein interactions. Moreover, further mutagenesis experiments suggested that the SSB and λExo binding sites may at least partially overlap.^48^ Though the CTD of Redβ was determined to be the main site of SSB peptide binding, the interaction only occurred with full‐length Redβ protein (i.e., the CTD of Redβ alone was insufficient). This suggests that both domains of Redβ were required for the interaction with SSB, whereas the CTD of Redβ is sufficient for binding to λExo.^24^ These findings from Bell and colleagues hint at an exciting secondary physiological role for the CTD of Redβ in SSA. Redβ may be targeted to the *E. coli* DNA replication fork through interaction of the CTD with SSB, which is then displaced from the ssDNA by the NTD of Red‐β. This model would explain the loss of recombination *in vivo* (see above) that was observed with C‐terminally mutated or truncated Redβ.^24^, ^25^

## 5 CURRENT MODELS FOR RECOMBINATION IN VIVO

**FIGURE 5:**
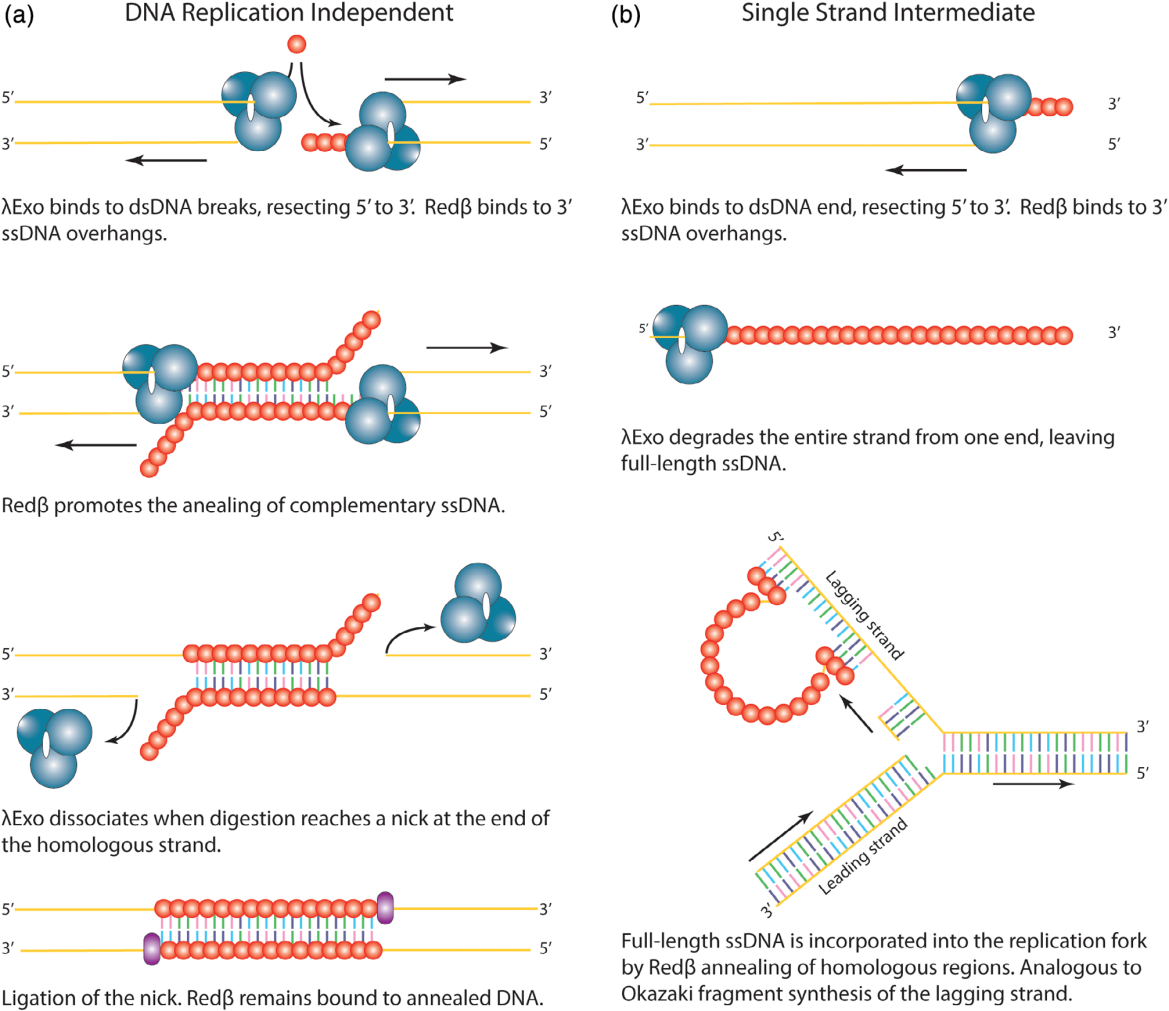
The two current models for SSA recombination. (a) Independent of a replication fork^51^; and (b) via a single‐stranded intermediate.^52^, ^53^ Redβ—red spheres; λExo—cyan spheres; ligase—purple ellipsoids. Figures are adapted from the references cited

The pioneering models for SSA, proposed in the early 1970s, lay the foundations for our current understanding of Red recombination. The single‐strand assimilation^13^, ^17^ and strand‐exchange models^17^ were λExo‐centric, pre‐dating the known function of Redβ by about a decade.^19^ Since then, these models have been continually evolving based on the work of many research groups. The accepted model for ssDNA recombination was proposed in 2002,^50^ but a consensus mechanism for dsDNA has been more difficult to achieve. There are two models for dsDNA recombination generally under consideration today: One that is independent of chromosomal DNA replication,^51^ and another that proceeds via a single‐stranded intermediate being incorporated at the replication fork^52^, ^53^ (Figure 5).

The first model proposes that two dsDNA ends are both processed by λExo, revealing 3′ ssDNA overhangs that contain homologous sequences. The two homologous regions are then annealed by Redβ (Figure 5a). The second model posits that the highly processive nature of λExo may often result in complete digestion of dsDNA into ssDNA products. This is supported by the complete hydrolysis of one strand of the phage λ chromosome, observed in single‐molecule experiments.^27^, ^31^ The full‐length ssDNA may then be incorporated at the lagging strand of the replication fork through Redβ‐mediated annealing between short regions of homology (Figure 5b).^52^, ^53^ The second model has the advantage of not requiring additional enzymes to resolve crossovers or fill gaps and provides the elegant solution of unifying the mechanisms for both ssDNA and dsDNA recombination. However, the two models are not necessarily mutually exclusive. It is possible that variably processed dsDNAs or ssDNA overhangs are recombined through different mechanisms.

## 6 AN EYE ON THE NEXT 50 YEARS

Despite more than half a century of research into the archetypal two‐component recombinase, there is work yet to be done. Where do we need to go next? What are the major gaps remaining in our knowledge and how do we bridge them?

As this review has demonstrated, λExo and Redβ have largely been studied in isolation, though they are known to exist in the same spatial and temporal context *in vivo*.^9^, ^10^ Therefore, many of the remaining questions center around the intricacies of how the two proteins function as a complex. A recent X‐ray crystal structure of λExo and the CTD of Redβ^48^ has begun to address this gap in knowledge. However, further research is required to build our understanding of the actions of the entire complex, and especially to reveal how trimeric λExo and dodecameric Redβ can possibly form a complex^39^ with the observed 1:1 subunit stoichiometry.^12^ In addition, the detailed molecular mechanism of how the broken substrate dsDNAs are processed and used in an SSA reaction remains murky. More experimentation is necessary to reveal the details of the processing and the intermediates formed, in order to put together a more comprehensive understanding of the underlying molecular mechanism.

This review has tracked the evolution of experimental approaches used to research phage λ recombination from initial ensemble biochemical characterization transitioning to single‐molecule and structural biology approaches, principally X‐ray crystallography. High‐resolution single particle reconstruction from cryo‐EM data is a technique that is now coming into its own. The challenges presented in structure determination of large and potentially dynamic complexes are well suited to this technique. Two decades ago, Passy *et al*. were able to show the potential of cryo‐EM in producing valuable insights into Redβ function.^39^ Since then, there have been major advances in the technologies surrounding cryo‐EM and the boundaries of what is possible will continue to be pushed (reviewed in Reference 54). From the ability to resolve multiple conformational states and the localized flexibility of complexes (available in software packages like RELION and CryoSparc v2.9) to the holy grail of in situ imaging of complexes in action, cryo‐EM is transforming the structural biology field.

The aim and the direction of our research are to answer the above‐mentioned questions regarding the phage λ Red system, using the various EM tools available, including cryo‐EM. The rapacity with which this technology and other tools of biochemical inquiry are evolving ensures that the next 50 years will see great advances in unraveling the molecular mechanisms underlying SSA.

## CONFLICT OF INTEREST

The authors declare no conflicts of interest.

## Supporting information


**Supplementary Animation 1** Non‐allelic breaks result in two DNA molecules (blue and red strands) that share a homologous region; 2—λExo (brown circles) binds to the dsDNA ends; 3 & 4—λExo digests 5′ to 3′ and Redβ annealase (green spheres) binds to the ssDNA overhangs generated by λExo activity; 5—Redβ catalyzes annealing of the homologous DNA sequences; 6 & 7—λExo stalls when it reaches the nick/gap in the DNA at the point of the original terminus, stopping digestion, and dissociating from the DNA.

## References

[1] Bhargava R , Onyango DO , Stark JM . Regulation of single‐strand annealing and its role in genome maintenance. Trends Genet. 2016;32:566–575. 27450436 10.1016/j.tig.2016.06.007 PMC4992407

[2] Weller SK , Sawitzke JA . Recombination promoted by DNA viruses: Phage λ to *Herpes simplex* virus. Annu Rev Microbiol. 2014;68:237–258. 25002096 10.1146/annurev-micro-091313-103424 PMC4303060

[3] Copeland NG , Jenkins NA , Court DL . Recombineering: A powerful new tool for mouse functional genomics. Nat Rev Genet. 2001;2:769–779. 11584293 10.1038/35093556

[4] Caldwell BJ , Bell CE . Structure and mechanism of the Red recombination system of bacteriophage λ. Prog Biophys Mol Biol. 2019;147:33–46. 30904699 10.1016/j.pbiomolbio.2019.03.005 PMC8054440

[5] Enquist LW , Skalka A . Replication of bacteriophage λ DNA dependent on the function of host and viral genes. I. Interaction of Red, Gam and Rec. J Mol Biol. 1973;75:185–212. 4580674 10.1016/0022-2836(73)90016-8

[6] Weissbach A , Korn D . The effect of lysogenic induction on the deoxyribonucleases of *Escherichia coli* K12 λ. J Biol Chem. 1962;237:3312–3314. 13999750

[7] Korn D , Weissbach A . The effect of lysogenic induction on the deoxyribonucleases of *Escherichia coli* K12‐λ. I. Appearance of a new exonuclease activity. J Biol Chem. 1963;238:3390–3394. 14085392

[8] Little JW , Lehman IR , Kaiser AD . An exonuclease induced by bacteriophage λ. I. Preparation of the crystalline enzyme. J Biol Chem. 1967;242:672–678. 6017736

[9] Radding CM . Regulation of λ exonuclease. I. Properties of λ exonuclease purified from lysogens of λ T11 and wild type. J Mol Biol. 1966;18:235–250. 5338754 10.1016/s0022-2836(66)80243-7

[10] Radding CM , Shreffler DC . Regulation of λ exonuclease. II. Joint regulation of exonuclease and a new λ antigen. J Mol Biol. 1966;18:251–261. 4961270 10.1016/s0022-2836(66)80244-9

[11] Little JW . An exonuclease induced by bacteriophage λ. II. Nature of the enzymatic reaction. J Biol Chem. 1967;242:679–686. 6017737

[12] Carter DM , Radding CM . The role of exonuclease and β protein of phage λ in genetic recombination. II. Substrate specificity and the mode of action of λ exonuclease. J Biol Chem. 1971;246:2502–2512. 4928646

[13] Radding CM , Carter DM . The role of exonuclease and β protein of phage λ in genetic recombination. III. Binding to deoxyribonucleic acid. J Biol Chem. 1971;246:2513–2518. 5553408

[14] Radding CM , Rosenweig J , Richards F , Cassuto E . Separation and characterisation of exonuclease, β protein, and a complex of both. J Biol Chem. 1971;246:2510–2512.

[15] Radding CM . The role of exonuclease and β protein of bacteriophage λ in genetic recombination. I. Effects of red mutants on protein structure. J Mol Biol. 1970;52:491–499. 4992641 10.1016/0022-2836(70)90415-8

[16] Shulman MJ , Hallick LM , Echols H , Signer ER . Properties of recombination‐deficient mutants of bacteriophage λ. J Mol Biol. 1970;52:501–520. 4923748 10.1016/0022-2836(70)90416-x

[17] Cassuto E , Radding CM . Mechanism for the action of λ exonuclease in genetic recombination. Nat New Biol. 1971;229:13–16. 5276049 10.1038/newbio229013a0

[18] Cassuto E , Lash T , Sriprakash KS , Radding CM . Role of exonuclease and β protein of phage λ in genetic recombination. V. Recombination of λ DNA in vitro. Proc Natl Acad Sci U S A. 1971;68:1639–1643. 4934524 10.1073/pnas.68.7.1639 PMC389258

[19] Kmiec E , Holloman WK . β protein of bacteriophage λ promotes renaturation of DNA. J Biol Chem. 1981;256:12636–12639. 6273399

[20] Muniyappa K , Radding CM . The homologous recombination system of phage λ. Pairing activities of β protein. J Biol Chem. 1986;261:7472–7478. 2940241

[21] Mythili E , Kumar KA , Muniyappa K . Characterisation of the DNA‐binding domain of β protein, a component of phage λ Red‐pathway, by UV catalyzed cross‐linking. Gene. 1996;182:81–87. 8982071 10.1016/s0378-1119(96)00518-5

[22] Karakousis G , Ye N , Li Z , Chiu SK , Reddy G , Radding CM . The β protein of phage λ binds preferentially to an intermediate in DNA renaturation. J Mol Biol. 1998;276:721–731. 9500924 10.1006/jmbi.1997.1573

[23] Sawitzke JA , Costantino N , Li XT , et al. Probing cellular processes with oligo‐mediated recombination and using the knowledge gained to optimise recombineering. J Mol Biol. 2011;407:45–59. 21256136 10.1016/j.jmb.2011.01.030 PMC3046259

[24] Smith CE , Bell CE . Domain structure of the Redβ single‐strand annealing protein: The C‐terminal domain is required for fine‐tuning DNA‐binding properties, interaction with the exonuclease partner, and recombination in vivo. J Mol Biol. 2016;428:561–578. 26780547 10.1016/j.jmb.2016.01.008

[25] Subramaniam S , Erler A , Fu J , et al. DNA annealing by Redβ is insufficient for homologous recombination and the additional requirements involve intra‐ and inter‐molecular interactions. Sci Rep. 2016;6:34525. 27708411 10.1038/srep34525 PMC5052646

[26] Dapprich J . Single‐molecule DNA digestion by λ‐exonuclease. Cytometry. 1999;36:163–168. 10404963 10.1002/(sici)1097-0320(19990701)36:3<163::aid-cyto3>3.0.co;2-r

[27] van Oijen AM , Blainey PC , Crampton DJ , Richardson CC , Ellenberger T , et al. Single‐molecule kinetics of λ exonuclease reveal base dependence and dynamic disorder. Science. 2003;301:1235–1238. 12947199 10.1126/science.1084387

[28] Subramanian K , Rutvisuttinunt W , Scott W , Myers RS . The enzymatic basis of processivity in λ exonuclease. Nucleic Acids Res. 2003;31:1585–1596. 12626699 10.1093/nar/gkg266 PMC152868

[29] Perkins TT , Dalal RV , Mitsis PG , Block SM . Sequence‐dependent pausing of single λ exonuclease molecules. Science. 2003;301:1914–1918. 12947034 10.1126/science.1088047 PMC1539570

[30] Matsuura S , Komatsu J , Hirano K , et al. Real‐time observation of a single DNA digestion by λ exonuclease under a fluorescence microscope field. Nucleic Acids Res. 2001;29:E79–E779. 11504887 10.1093/nar/29.16.e79 PMC55863

[31] Oliver‐Calixte NJ , Uba FI , Battle KN , Weerakoon‐Ratnayake KM , Soper SA . Immobilisation of λ exonuclease onto polymer micropillar arrays for the solid‐phase digestion of dsDNAs. Anal Chem. 2014;86:4447–4454. 24628008 10.1021/ac5002965 PMC4018173

[32] Lee G , Yoo J , Leslie BJ , Ha T . Single‐molecule analysis reveals three phases of DNA degradation by an exonuclease. Nat Chem Biol. 2011;7:367–374. 21552271 10.1038/nchembio.561 PMC3097319

[33] Stahl FW , McMilin KD , Stahl MM , Nozu Y . An enhancing role for DNA synthesis in formation of bacteriophage λ recombinants. Proc Natl Acad Sci U S A. 1972;69:3598–3601. 4509320 10.1073/pnas.69.12.3598 PMC389829

[34] van Oostrum J , White JL , Burnett RM . Isolation and crystallisation of λ exonuclease. Arch Biochem Biophys. 1985;243:332–337. 2935081 10.1016/0003-9861(85)90510-7

[35] Kovall R , Matthews BW . Toroidal structure of λ exonuclease. Science. 1997;277:1824–1827. 9295273 10.1126/science.277.5333.1824

[36] Zhang J , McCabe KA , Bell CE . Crystal structures of λ exonuclease in complex with DNA suggest an electrostatic ratchet mechanism for processivity. Proc Natl Acad Sci U S A. 2011;108:11872–11877. 21730170 10.1073/pnas.1103467108 PMC3141983

[37] Pan X , Smith CE , Zhang J , McCabe KA , Fu J , Bell CE . A structure‐activity analysis for probing the mechanism of processive double‐stranded DNA digestion by λ exonuclease trimers. Biochemistry. 2015;54:6139–6148. 26361255 10.1021/acs.biochem.5b00707

[38] Pan X , Yan J , Patel A , Wysocki VH , Bell CE . Mutant poisoning demonstrates a nonsequential mechanism for digestion of double‐stranded DNA by λ exonuclease trimers. Biochemistry. 2015;54:942–951. 25531139 10.1021/bi501431w

[39] Passy SI , Yu X , Li Z , Radding CM , Egelman EH . Rings and filaments of β protein from bacteriophage λ suggest a superfamily of recombination proteins. Proc Natl Acad Sci U S A. 1999;96:4279–4284. 10200253 10.1073/pnas.96.8.4279 PMC16323

[40] Poteete AR , Sauer RT , Hendrix RW . Domain structure and quaternary organisation of the bacteriophage p22 Erf protein. J Mol Biol. 1983;171:401–418. 6607360 10.1016/0022-2836(83)90037-2

[41] Thresher RJ , Makhov AM , Hall SD , Kolodner R , Griffith JD . Electron‐microscopic visualisation of RecT protein and its complexes with DNA. J Mol Biol. 1995;254:364–371. 7490755 10.1006/jmbi.1995.0623

[42] Shinohara A , Shinohara M , Ohta T , Matsuda S , Ogawa T . Rad52 forms ring structures and co‐operates with RPA in single‐strand DNA annealing. Genes Cells. 1998;3:145–156. 9619627 10.1046/j.1365-2443.1998.00176.x

[43] Van Dyck E , Hajibagheri NMA , Stasiak A , West SC . Visualisation of human Rad52 protein and its complexes with hRad51 and DNA. J Mol Biol. 1998;284:1027–1038. 9837724 10.1006/jmbi.1998.2203

[44] Tolun G , Makhov AM , Ludtke SJ , Griffith JD . Details of ssDNA annealing revealed by an HSV‐1 ICP8‐ssDNA binary complex. Nucleic Acids Res. 2013;41:5927–5937. 23605044 10.1093/nar/gkt266 PMC3675482

[45] Saotome M , Saito K , Yasuda T , et al. Structural basis of homology‐directed DNA repair mediated by Rad52. iScience. 2018;3:50–62. 30428330 10.1016/j.isci.2018.04.005 PMC6137706

[46] Erler A , Wegmann S , Elie‐Caille C , et al. Conformational adaptability of Redβ during DNA annealing and implications for its structural relationship with Rad52. J Mol Biol. 2009;391:586–598. 19527729 10.1016/j.jmb.2009.06.030

[47] Ander M , Subramaniam S , Fahmy K , Stewart AF , Schaffer E . A single‐strand annealing protein clamps DNA to detect and secure homology. PLoS Biol. 2015;13:e1002213. 26271032 10.1371/journal.pbio.1002213 PMC4535883

[48] Caldwell BJ , Zakharova E , Filsinger GT , et al. Crystal structure of the Redβ C‐terminal domain in complex with λ exonuclease reveals an unexpected homology with λ Orf and an interaction with *Escherichia coli* single stranded DNA binding protein. Nucleic Acids Res. 2019;47:1950–1963. 30624736 10.1093/nar/gky1309 PMC6393309

[49] Shereda RD , Kozlov AG , Lohman TM , Cox MM , Keck JL . SSB as an organiser/mobiliser of genome maintenance complexes. Crit Rev Biochem Mol Biol. 2008;43:289–318. 18937104 10.1080/10409230802341296 PMC2583361

[50] Court DL , Sawitzke JA , Thomason LC . Genetic engineering using homologous recombination. Annu Rev Genet. 2002;36:361–388. 12429697 10.1146/annurev.genet.36.061102.093104

[51] Lin FL , Sperle K , Sternberg N . Model for homologous recombination during transfer of DNA into mouse L cells: Role for DNA ends in the recombination process. Mol Cell Biol. 1984;4:1020–1034. 6330525 10.1128/mcb.4.6.1020-1034.1984 PMC368869

[52] Mosberg JA , Lajoie MJ , Church GM . λ Red recombineering in *Escherichia coli* occurs through a fully single‐stranded intermediate. Genetics. 2010;186:791–799. 20813883 10.1534/genetics.110.120782 PMC2975298

[53] Maresca M , Erler A , Fu J , Friedrich A , Zhang YM , et al. Single‐stranded heteroduplex intermediates in λ Red homologous recombination. BMC Mol Biol. 2010;11:54. 20670401 10.1186/1471-2199-11-54 PMC2918612

[54] Cheng YF . Single‐particle cryo‐EM—How did it get here and where will it go. Science. 2018;361:876–880. 30166484 10.1126/science.aat4346 PMC6460916

